# A generative artificial intelligence approach for peptide antibiotic optimization

**DOI:** 10.1038/s42256-026-01237-5

**Published:** 2026-05-13

**Authors:** Marcelo D. T. Torres, Yimeng Zeng, Fangping Wan, Natalie Maus, Jacob Gardner, Cesar de la Fuente-Nunez

**Affiliations:** 1 Machine Biology Group, Departments of Psychiatry and Microbiology, Institute for Biomedical Informatics, Institute for Translational Medicine and Therapeutics, Perelman School of Medicine, University of Pennsylvania, Philadelphia, PA USA; 2 Departments of Bioengineering and Chemical and Biomolecular Engineering, School of Engineering and Applied Science, University of Pennsylvania, Philadelphia, PA USA; 3 Department of Chemistry, School of Arts and Sciences, University of Pennsylvania, Philadelphia, PA USA; 4 Penn Institute for Computational Science, University of Pennsylvania, Philadelphia, PA USA; 5 Department of Computer and Information Science, University of Pennsylvania, Philadelphia, PA USA

**Keywords:** Computational biology and bioinformatics, Microbiology

## Abstract

Antibiotic resistance is rising globally, demanding faster, more reliable routes to design antimicrobial candidates. Although artificial-intelligence-based methods have accelerated antimicrobial discovery, most are designed to screen fixed libraries or generate candidates broadly, rather than optimize existing peptide scaffolds under practical design constraints. Here, to address this challenge, we present APEX generative optimization (ApexGO). ApexGO uses a transformer variational autoencoder that embeds peptide sequences in a continuous latent space, whereas Bayesian optimization efficiently proposes sequence edits to boost antimicrobial potency. Unlike traditional approaches, ApexGO generates peptide sequences through modifications of template peptides, opening avenues for peptide design and antibiotic discovery. Using ten peptides as templates, ApexGO generated optimized derivatives with enhanced antimicrobial properties. We chemically synthesized 100 of these compounds and conducted comprehensive in vitro characterizations, including assessments of antimicrobial activity, mechanism of action, secondary structure and cytotoxicity. In particular, ApexGO achieved an 85% ground-truth experimental hit rate and a 72% success rate in enhancing antimicrobial activity against Gram-negative pathogens, outperforming previously reported methods for antibiotic discovery and optimization. In two preclinical mouse models of *Acinetobacter baumannii* infection, artificial-intelligence-optimized molecules exhibited potent anti-infective activity superior to their template controls and comparable with or exceeding that of last-resort antibiotic. These findings highlight the potential of ApexGO as a generative artificial intelligence approach for peptide design and antibiotic optimization, offering a powerful tool to accelerate antibiotic discovery.

## Abstract (web summary)

Torres et al. present ApexGO, a generative approach capable of redesigning peptide antibiotics to better kill drug-resistant bacteria. They validated candidates in laboratory tests and mouse infections and matching or outperforming standard antibiotics.

## Main

Antimicrobial resistance (AMR) poses a substantial and escalating global health threat, necessitating the discovery of antibiotics to combat resistant pathogens^1^. Although most clinically used antibiotics are small molecules, peptide antibiotics^1–3^ such as polymyxin B and colistin have become critical last-line therapies against multidrug-resistant Gram-negative pathogens, including *Acinetobacter baumannii*, *Klebsiella pneumoniae* and *Pseudomonas aeruginosa*. However, resistance to polymyxins is now increasingly reported^4,5^, threatening the efficacy of these essential agents. At the same time, the development of peptide antibiotics remains challenging^6^ due to the vastness of the peptide sequence space and the limited understanding of how specific sequence features affect antimicrobial potency. Importantly, optimizing these molecules experimentally remains exceedingly challenging as it relies of painstaking trial-and-error approaches. These challenges underscore the need for optimization strategies to improve the efficacy of peptide antibiotics and expand the repertoire of available scaffolds.

Recent advances in molecular de-extinction^7,8^ have opened alternative avenues for antibiotic discovery by identifying and characterizing biological molecules from the past. This approach has uncovered an untapped sequence space that may harbour potent antimicrobial agents capable of fighting modern challenges like AMR. Leveraging this concept, we previously developed APEX^8^, a deep learning-based functional predictor of antimicrobial activity, to mine all extinct organisms known to science, leading to the discovery of numerous antibiotics.

Computational pipelines for antimicrobial peptide (AMP) discovery and design span several complementary strategies^6,9^. Curated AMP databases and genome-mining efforts provide rich starting libraries, which can be prioritized using supervised machine learning predictors that screen large sequence libraries in silico^10–15^. More recently, deep generative models—including latent-variable and diffusion-based approaches—have been used to propose de novo AMP candidates conditioned on broad properties^16–18^. Although these approaches are powerful for identifying promising starting points, they typically do not directly solve the lead optimization setting: improving a given scaffold to meet a target activity profile and preserving practical constraints such as similarity to a parent peptide, bounded edit distances or manufacturability.

Accordingly, even after a candidate peptide has been identified, optimizing its antimicrobial profile under realistic constraints remains challenging. Traditional methods rely heavily on iterative experimental modifications, which are time-consuming and resource-intensive. This motivates computational optimization methods that can efficiently navigate local sequence neighbourhoods and trade off potency, spectrum of activity and other properties, as well as enforce user-defined constraints.

In this study, we introduce APEX generative optimization (ApexGO), a generative artificial intelligence (AI) framework that integrates deep generative modelling with Bayesian optimization (BO) to streamline peptide design and optimization. ApexGO uses a transformer-based variational autoencoder (VAE) to map peptide sequences into a continuous latent space, transforming the discrete optimization problem into a tractable continuous one. Here we trained a generative VAE model for peptide sequences. By incorporating BO techniques, including our own methods like local latent Bayesian optimization (LOL-BO)^19^, ApexGO efficiently explores this latent space to identify peptides with enhanced antimicrobial properties. Combining generative AI and BO represents a substantial methodological departure from recent successful prior work that uses supervised deep learning to virtually screen large but fixed databases of molecules^13–15^. ApexGO instead searches over arbitrary peptide modifications and can suggest peptides that would not exist in any known database. Our work takes a fundamentally different approach to peptide design by framing it as an optimization problem, rather than relying on one-shot generation from a generative model. Unlike standard generative modelling approaches, BO incorporates feedback in a closed-loop fashion—when generating peptide *t*, the observed scores of peptides $1\ldots t-1$ are used to guide the next step in the design process. Different from prior work^19,20^ in generative BO, we seek to design optimized derivatives of existing templates. We augment the latent space BO with design and diversity constraints, and a per-template fine-tuned peptide VAE. In particular, our work represents the first ground-truth in vitro or in vivo experimental validation of generative BO, and provides an alternative for optimizing the antibiotic function of peptides.

Using ten de-extinct peptides as templates, we applied ApexGO to generate optimized derivatives with improved antimicrobial activity (Supplementary Table 1). The templates were selected based on their varied activity profiles and potential for enhancement. We synthesized 100 optimized peptides and conducted comprehensive in vitro characterizations, including the assessments of antimicrobial efficacy against clinically relevant pathogens, mechanism of action studies, secondary structure analyses and cytotoxicity evaluations.

In particular, the optimized peptides demonstrated significant improvements in antimicrobial potency, including activity against multidrug-resistant strains. In preclinical mouse models of *A. baumannii* infection, several optimized molecules—specifically those derived from mammuthusin-3 and mylodonin-2—exhibited potent anti-infective activity comparable with or exceeding that of polymyxin B, a last-resort antibiotic. These findings highlight the effectiveness of ApexGO as a generative AI approach for antibiotic optimization, offering a potential path forward in tackling AMR.

## Results and discussion

### ApexGO: integrating deep learning with BO

We recently developed APEX, a deep learning approach to predict antibiotic function from amino acid sequence^8^. APEX efficiently predicts the minimal inhibitory concentrations (MICs) of peptides against a variety of Gram-negative and Gram-positive bacterial pathogens (see the ‘APEX 1.1’ section). Although APEX successfully discovered antibiotic molecules from extinct organisms, its discriminative learning nature limits its capacity for de novo design or property optimization. Moreover, the vastness of the peptide sequence space renders exhaustive virtual screening infeasible.

**Fig. 1:**
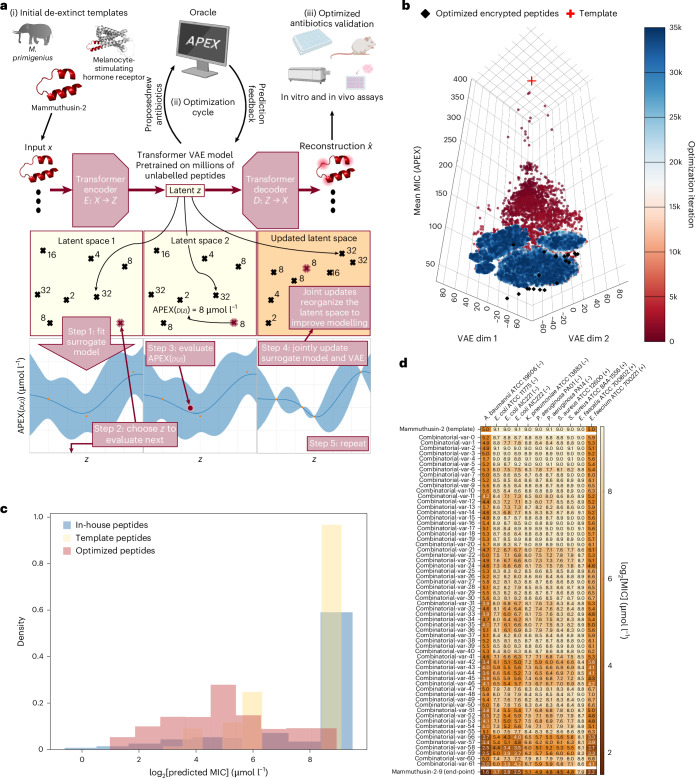
ApexGO: computational workflow and results. **a**, Overall workflow of ApexGO demonstrates de-extinction, optimization and validation. The workflow shows how the peptide VAE is jointly updated with the surrogate model during optimization. **b**, Visualization of latent space optimization in two dimensions using *t*-distributed stochastic neighbour embedding. The red cross indicates the template peptide sequence with a high APEX-predicted mean MIC. The black diamonds represent the optimized peptides identified by ApexGO. Scattered points correspond to latent space samples evaluated during optimization, with the colour gradient indicating the progression of optimization iterations from early (red) to later stages (blue). **c**, Comparison of log_2_ transformed MIC predictions by APEX for three peptide groups: the in-house peptides used to train APEX, the ten peptide templates selected for optimization and the optimized peptides generated by ApexGO. Histograms display the distribution of predicted MIC values, demonstrating a notable shift towards lower MICs in the optimized peptides, indicative of enhanced predicted antimicrobial activity. **d**, Heat map showing the predicted MICs for a specific template peptide (top row), the final optimized peptide (bottom row) and intermediate peptide variants (middle rows) obtained by systematically reverting individual amino acid substitutions back to the template sequence. This analysis illustrates that the amino acid changes introduced by ApexGO are computationally necessary for improving the antimicrobial potential. Illustration in **a** created in BioRender; de la Fuente-Nunez, C. http://biorender.com/i1jrfz3 (2026).

To address these challenges, we developed ApexGO, which integrates APEX with black-box BO and generative modelling methods to perform constrained template-based optimization of antibiotic activity in peptide sequences (Fig. 1a). ApexGO uses a transformer-based VAE^21,22^ to map the peptide sequences into a continuous latent space, effectively transforming the discrete optimization problem into a tractable continuous one. This approach enables the efficient exploration of the sequence space to identify molecules with improved antimicrobial properties.

In ApexGO, the generation process involves sampling latent space points and decoding them into peptide sequences using the VAE decoder. The evaluation process uses APEX to predict the antimicrobial potential of the generated peptides. A surrogate model, implemented as a parametric Gaussian process regressor^23^, models the correlation between the latent space points and the APEX-predicted MICs. The BO algorithm utilizes this surrogate model to propose latent space points probably to decode into peptides with enhanced antimicrobial activity. This iterative process continues until peptides with optimized properties are identified.

Key features of ApexGO include the following.

1. BO over adaptive latent spaces: by training a VAE that maps peptides to a continuous latent space, we convert a discrete optimization problem into a continuous one, facilitating optimization. Periodic joint updates of the VAE and surrogate model encourage the latent space to reorganize, clustering peptides with similar MICs closer together, thereby enhancing modelling and optimization.
2. Multiple trust regions: to prevent over-exploration in the high-dimensional latent space, we implement a trust region BO^24^. This method defines hyper-rectangular regions centred on the best points observed so far, adapting their size based on optimization progress. By using multiple trust regions, we simultaneously optimize multiple distinct peptides in a single run.

### Template-based AMP design and optimization

In this work, we focused on template-based AMP optimization, using ten peptides mined from the proteomes of extinct organisms as templates. These templates were selected based on their varied activity profiles, ranging from selective to broad-spectrum effects. Importantly, none of the template de-extinct peptides were highly active (MIC ≤ 16 μmol l^−1^), ensuring room for 4- to 32-fold potency gains (Supplementary Data 1). We intentionally focused on a starting set of peptides with mid-micromolar MICs to give ApexGO headroom to demonstrate meaningful improvements, and better simulate the typical setting of lead development. Starting from nanomolar-active peptides would (1) collapse this dynamic (at such low concentrations, assay noise can be comparable with or exceed true improvements) and (2) require the APEX oracle to extrapolate well beyond the range of peptides it was trained on, rendering its predictions less accurate. Additionally, for most membrane-related or non-specific mechanisms, peptides must reach a threshold concentration to exhibit activity unless they are receptor mediated, which were not included in APEX’s training and were, therefore, excluded from our optimization. ApexGO performed constrained peptide sequence optimization, ensuring that each optimized sequence maintained at least 75% sequence similarity to its template (Methods). The optimization aimed to improve antimicrobial activities against either seven Gram-negative pathogen strains or all 11 strains (including Gram-positive bacteria) that APEX predicts (see the ‘APEX 1.1’ section).

Starting from the selected template sequences with relatively high average MICs against Gram-negative bacteria, ApexGO generated sequences with progressively lower predicted MICs as optimization progressed (Fig. 1b). ApexGO was tasked to produce ten optimized peptides from each of the ten starting templates, for a total of 100 product peptides, of which we will go on to experimentally validate all of them to avoid and characterize any potential selection bias. Compared with the MIC distributions of the APEX training peptides and the template peptides, the optimized peptides showed a substantial shift towards lower experimental MICs, indicating enhanced predicted antimicrobial activities (Fig. 1c). Statistical analysis confirmed that the prediction improvements were significant (*P* values of one-sided Mann–Whitney *U*-test were 1.19 × 10^−309^ and 1.12 × 10^−35^ when comparing optimized peptides with in-house and template peptides, respectively).

To assess the contribution of each amino acid change proposed by ApexGO, we generated intermediate variants by systematically reverting individual mutations in the optimized sequences back to the template amino acids (Fig. 1d). By evaluating these variants with APEX and ranking them based on the predicted MICs, we found that the optimized peptides were almost always ranked first (mean ranking of 1.23; standard deviation of 0.42), suggesting that each substitution contributed incrementally to the predicted antimicrobial improvement according to APEX; however, these insights are based on computational evaluation and were not experimentally confirmed.

To benchmark ApexGO against other deep generative AMP frameworks, we directly compared it with HydrAMP^16^ and the PepDiffusion^17^ latent diffusion model on the same template-constrained optimization task. Using the official HydrAMP implementation and generator/decomposer weights, we generated analogues for each of the ten extinct peptide templates under the ‘discovery’ criterion at decoder temperatures of *T* = 3, 5 and 10, applied the same ≥75% sequence-similarity constraint used in our main experiments, and scored all feasible candidates with the APEX Gram-negative objective (Supplementary Fig. 1). Across all seeds and temperatures, the best feasible HydrAMP analogues consistently exhibited higher (less-active) APEX-predicted MIC values than the corresponding ApexGO designs, and only 22.2%, 10.8% and 0.2% of HydrAMP proposals at *T* = 3, 5 and 10, respectively, satisfied the similarity threshold, with no feasible analogue obtained for one seed at *T* = 10 even after exhausting the full proposal budget. By contrast, when we used PepDiffusion with the study’s best-performing checkpoints and default sampling hyperparameters to generate 10,000 AMP-conditioned sequences (9,962 unique), none of the candidates met the ≥75% similarity threshold to any of the ten templates, preventing a meaningful objective comparison. Together, these benchmarks indicate that although HydrAMP and PepDiffusion are well suited for unconstrained, diversity-oriented AMP discovery, they either produce few or no viable candidates when repurposed for per-template, similarity-constrained optimization, whereas ApexGO reliably identifies high-scoring derivatives under these design constraints.

#### Ground-truth in vitro antimicrobial activity of optimized peptides

**Fig. 2:**
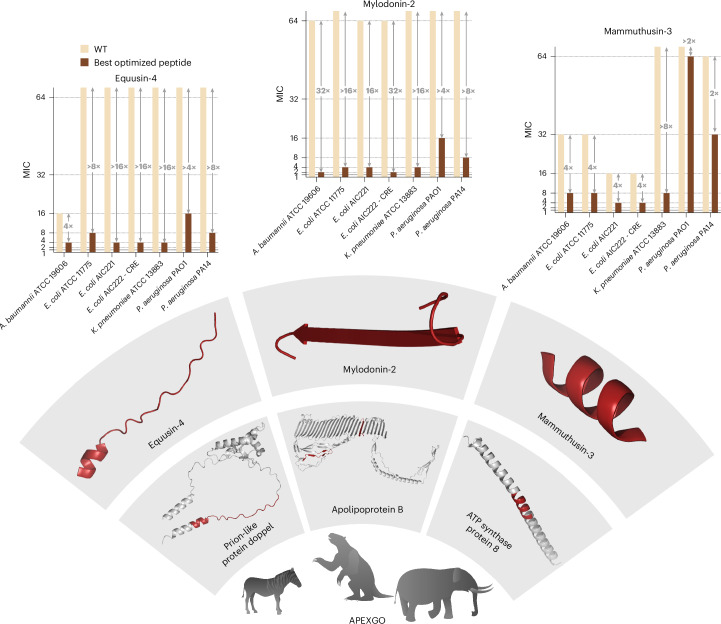
Antimicrobial activity of peptides optimized by ApexGO. Heat map displaying the MIC (in μmol l^−^^1^) of peptides optimized by ApexGO against clinically relevant bacterial pathogens. The MICs were determined using the broth microdilution method, and the bar plots present the mode MIC values from three independent biological replicates obtained for the best-performing optimized peptide for each of the templates. The optimized peptides demonstrate notable improvements in antimicrobial efficacy compared with their parent templates, validating the effectiveness of ApexGO in generating peptides with enhanced activity against bacterial strains. WT, wild type.

To validate the predictive accuracy of ApexGO, we synthesized and tested two sets of optimized peptides per de-extinct template: (1) five peptides predicted to be active against Gram-negative bacteria and (2) five peptides predicted to be broad spectrum. ApexGO produced a total of 100 optimized peptides, which were synthesized and experimentally tested for antimicrobial activity against 11 clinically relevant bacterial pathogens, including strains resistant to conventional antibiotics^25–27^ (Fig. 2 and Supplementary Fig. 2). Although ApexGO produces a much larger number of intermediate peptides during optimization and running to produce the final optimized peptides, the experimental performance of these intermediate peptides is ultimately related to the rank correlation of the APEX model itself, not to the ApexGO procedure introduced here.

Of the 100 synthesized peptides, 86 exhibited detectable antimicrobial activity (MIC ≤ 64 μmol l^−1^) against at least one bacterial strain, resulting in an 86% hit rate. Pearson and Spearman correlation coefficients between experimentally determined MICs and APEX predictions for the 100 optimized sequences were 0.463 and 0.462, respectively, underscoring APEX’s predictive power and the effectiveness of using APEX as the oracle for BO.

Comparing the mean experimentally determined MICs between optimized peptides and their corresponding templates, we observed that 68% of the optimized peptides showed improved antimicrobial activity after optimization with ApexGO (Fig. 2). When considering the Gram-negative strains only, the improvement rate reached 72%. Template-wise and bacterial-strain-wise analyses (that is, in terms of average mean MIC improvement rate and average MIC log-fold change) indicated that ApexGO was particularly effective at optimizing peptides against Gram-negative pathogens (85% hit rate), consistent with APEX’s stronger predictive performance for these bacteria^8^. That being said, peptides optimized for broad-spectrum activity had significantly lower MIC distributions than those optimized specifically for Gram-negative bacteria (*P* value of one-sided Mann–Whitney *U*-test, 0.006), highlighting the value of broad-spectrum optimization in ApexGO.

Specific observations include modifications to equusin-4 derivatives, such as replacing histidine with isoleucine at position 18 (H18I), which improved activity against Gram-negative strains, particularly *A. baumannii*, *Escherichia coli* and *P. aeruginosa*. Arctoterin-1 optimized peptides showed increased activity against Gram-negative bacteria, with substitutions such as histidine to glycine at position 6 (H6G) enhancing activity against *S. aureus*. Positively charged modifications in lophiosin-1 at the N terminus maintained antimicrobial activity, whereas negatively charged substitutions failed to improve the broad-spectrum efficacy. In mylodonin-2, substitutions like threonine to arginine or tryptophan at position 10 (T10R or T10W) enhanced activity against *K. pneumoniae*, especially when combined with amphiphilic N-terminal modifications. For mammuthusins 2 and 3 peptides, increases in normalized hydrophobic moment generally correlated with improved activity, except in specific cases in which crucial residues were altered, such as isoleucine at position 6 in mammuthusin-3-5. Hydrodamin-2 peptides showed increased or maintained activity with various modifications, except for hydrodamin-2-10, which had a unique tyrosine to tryptophan substitution at position 9 (Y9W). Finally, hesperelin-3 peptides lost activity with the introduction of a proline residue in place of glycine at position 6 (G6P), whereas the most effective broad-spectrum peptides preserved the arginine at position 1 (R1).

Many of the most potent ApexGO-optimized peptides featured lysine substitutions or insertions (Supplementary Table 1), a modification previously shown to promote Gram-negative accumulation. This observation aligns with findings from small-molecule studies, where the addition of primary amines notably improved their uptake by *E. coli*^28^. Although our work focuses on peptides rather than small molecules, this convergence—through increased cationic character—suggests a potentially generalizable strategy for enhancing Gram-negative permeability across diverse compound classes.

#### Secondary structure of optimized peptides

**Fig. 3:**
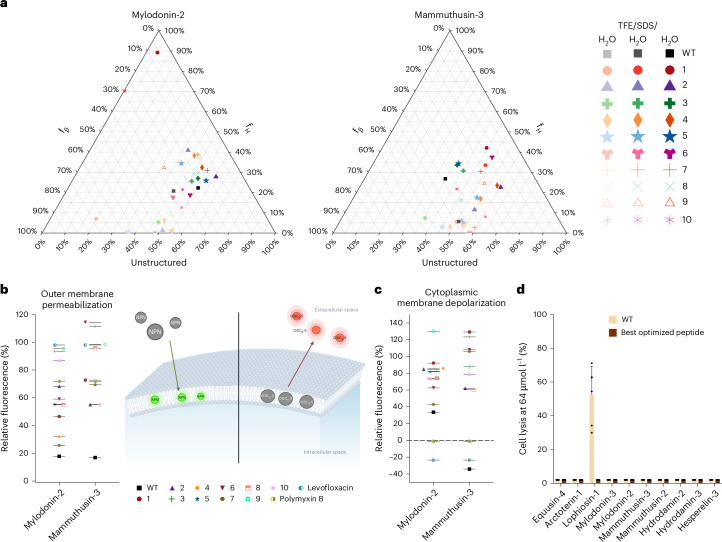
Secondary structure, cytotoxicity and mechanism of action of templates and peptides optimized by ApexGO. **a**, Ternary plots showing the percentage of secondary structure for two peptide families (mylodonin-2 and mammuthusin-3) at 50 μmol l^−1^ in three different solvents: water, 60% TFE in water and SDS (10 mmol l^−1^) in water. Secondary structure fractions were calculated using the BeStSel server^56^. **b**, Outer membrane permeabilization of *A. baumannii* ATCC 19606 induced by the peptides, assessed using the fluorescent probe NPN. The values represent the end-point relative fluorescence units (RFU) obtained after nonlinear fitting, compared with the baseline of the untreated control (buffer + bacteria + NPN), and benchmarked against the template peptides and the antibiotics polymyxin B and levofloxacin. **c**, Cytoplasmic membrane depolarization of *A. baumannii* ATCC 19606 induced by the peptides, evaluated using the potentiometric fluorescent probe DiSC_3_-5. The values displayed represent the end-point RFU obtained after nonlinear fitting, compared with the baseline of the untreated control (buffer + bacteria + DiSC_3_-5), and benchmarked against the template peptides and the antibiotics polymyxin B and levofloxacin. **d**, Cytotoxicity was assessed by exposing human embryonic kidney (HEK293T) cells to the peptides at 64 μmol l^−1^, the highest concentration used in the antimicrobial activity assays, and comparing the effects of the original templates with those of the selected optimized peptides. All experiments were performed in three independent replicates. Cytotoxicity experiments were made with two technical replicates per independent replicate. Illustration in **b** created in BioRender; de la Fuente-Nunez, C. http://biorender.com/avnmqwk (2026).

Since the optimized peptides sometimes differed substantially from the templates in terms of physicochemical descriptors and antimicrobial activity profiles, we investigated whether the optimization led to changes in their secondary structure compared with the templates. To assess the secondary structure of the peptides, we exposed them to three different media (Fig. 3a and Supplementary Figs. 3–5): water, helix-inducing medium^29^ (trifluoroethanol (TFE) in water, 3:2, v:v) and membrane-mimicking environment (sodium dodecyl sulfate (SDS) at 10 mmol l^−1^).

Equusin-4 derivatives exhibiting higher helicity (Supplementary Figs. 3a–c and 5), particularly in the helix-inducing medium, were more active, especially against *K. pneumoniae* (Fig. 2). By contrast, arctoterin-1 derivatives (Supplementary Figs. 3d–f and 5) showed no clear correlation between helicity and activity; the optimized peptides that were more active against Gram-negative bacteria were less helical. Lophiosin-1 (Supplementary Figs. 3g–i and 5) and mylodonin-2 (Fig. 3a and Supplementary Figs. 3m–o and 5) derivatives were mostly β-structured or unstructured. In these cases, lower helicity correlated with higher activity, whereas more α-helical derivatives were inactive. Although mylodonin-3 derivatives were primarily unstructured, peptides with slightly higher β-sheet content were more active (Supplementary Figs. 3j–l and 5).

Mammuthusin-2 (Supplementary Figs. 4d–f and 5) and mammuthusin-3 (Fig. 3a and Supplementary Figs. 4a–c and 5) derivatives exhibited low helicity with a slight increase in the α-helical structure in lipid bilayers; however, no clear correlation with antimicrobial activity was observed. Similarly, hydrodamin-2 (Supplementary Figs. 4g–i and 5) and hydrodamin-3 (Supplementary Figs. 4j–l and 5) derivatives showed a slight increase in α-helicity in membrane-mimicking environments, but their antimicrobial activity did not correlate with the secondary structure. Hesperelin-3 derivatives were mostly unstructured (Supplementary Figs. 4m–o and 5). An exception was one derivative that showed an increase in α-helicity is the presence of lipid bilayers; however, this did not strongly correlate with activity. Overall, no consistent trend was observed between the secondary structure and the antimicrobial activity across the peptides studied. These findings illustrate that ApexGO-generated peptides can adopt a wide range of structural conformations—α-helical, β-like or disordered—highlighting that antimicrobial activity can emerge from diverse structural backgrounds, and that potency improvements are not strictly dependent on converging towards a specific secondary structure.

#### Mechanism of action of optimized peptides

The bacterial membrane is a common target for most AMPs, where they engage in non-specific interactions with the lipid bilayer^30^. The antimicrobial activity of AMPs is influenced by their amino acid composition, distribution and various physicochemical characteristics such as amphiphilicity and hydrophobicity. To investigate the underlying mechanisms by which the AI-optimized peptides predicted by ApexGO kill bacteria, we tested whether differences in the composition of the derivatives—resulting from substitutions and/or insertions made to the original sequences—would affect their mechanism of action. For these assays, we selected *A. baumannii* ATCC 19606, which was the most sensitive strain in our MIC assays.

First, we tested whether the peptides permeabilized the bacterial outer membrane using 1-(*N*-phenylamino)naphthalene (NPN) assays (Fig. 3b and Supplementary Fig. 6). NPN, a lipophilic dye, faintly fluoresces in aqueous solutions but fluoresces more when it encounters lipidic environments such as bacterial membranes. NPN can penetrate the bacterial outer membrane only if it is disrupted or compromised. Among the mylodonin-3 derivatives, mylodonin-3-7 was the only analogue that exhibited superior permeabilization compared with both control antibiotics (Supplementary Fig. 6g,h). Mammuthusin-3 derivatives mammuthusin-3-3, mammuthusin-3-8 and mammuthusin-3-9 also showed slightly better permeabilization than the controls (Fig. 3b and Supplementary Fig. 6k,l), although not as effective as mylodonin-3-7. Within the mammuthusin-2 family, mammuthusin-2-7 had the highest permeabilization activity, with derivatives mammuthusin-2-2, mammuthusin-2-8, mammuthusin-2-9 and mammuthusin-2-10 being slightly more effective than the antibiotics (Supplementary Fig. 6m,n). Arctoterin-1-9 was the most effective permeabilizer among its family, whereas derivatives arctoterin-1-3 and arctoterin-1-7 demonstrated slight permeabilization improvements over the controls (Supplementary Fig. 6c,d) Equusin-4-7 was the strongest permeabilizer among its derivatives, with equusin-4-10 showing marginally better depolarization than the antibiotics (Supplementary Fig. 6a,b). By contrast, the only lophiosin-3 derivative active against *A. baumannii* was not an effective permeabilizer (Supplementary Fig. 6e,f). Additionally, none of the mylodonin-2 (Fig. 3b and Supplementary Fig. 6i,j), hesperelin-3 (Supplementary Fig. 6s,t), hydrodamin-3 (Supplementary Fig. 6q,r) or hydrodamin-2 (Supplementary Fig. 6o,p) derivatives demonstrated notable permeabilization activity.

Next, we tested whether the optimized peptides depolarized the cytoplasmic membrane of *A. baumannii* (Fig. 3c and Supplementary Fig. 7). We used the potentiometric fluorophore 3,3′-dipropylthiadicarbocyanine iodide (DiSC_3_-5), whose fluorescence is suppressed by its accumulation and aggregation within the cytoplasmic membrane. On disturbances in the transmembrane potential of the cytoplasmic membrane, this fluorophore migrates to the outer environment and emits fluorescence. Polymyxin B was used as a positive control in these experiments, as it is a depolarizer that also permeabilizes and damages bacterial membranes. All the tested peptides demonstrated stronger depolarization activity compared with the antibiotics polymyxin B and levofloxacin. In this work, polymyxin B and levofloxacin are used solely as positive controls to contextualize effect against bacterial pathogens; mechanistic class, macrocyclization and pharmacokinetic differences preclude a direct comparison of these antibiotics with our linear peptides. Among the mylodonin-3 derivatives, mylodonin-3-7 was the most effective depolarizer of *A. baumannii*, followed closely by mylodonin-3-3, mylodonin-3-4, mylodonin-3-6, mylodonin-3-8 and mylodonin-3-10 (Supplementary Fig. 7g,h). Mylodonin-2-9 was the top depolarizer among the mylodonin-2 analogues (Fig. 3c and Supplementary Fig. 7i,j). Mammuthusin-3 derivatives mammuthusin-3-1, mammuthusin-3-3, mammuthusin-3-6 and mammuthusin-3-7 were slightly better depolarizers than the others (Fig. 3c and Supplementary Fig. 7k,l), whereas mammuthusin-2-7 exhibited the highest depolarization activity within its family, followed by derivatives mammuthusin-2-2, mammuthusin-2-3, mammuthusin-2-8, mammuthusin-2-9 and mammuthusin-2-10 (Supplementary Fig. 7m,n). Lophiosin-1-3 (Supplementary Fig. 7e,f) and several hydrodamin-3 analogues (hydrodamin-3-6, hydrodamin-3-7, hydrodamin-3-9 and hydrodamin-3-10) were also effective depolarizers (Supplementary Fig. 7q,r). Among the hydrodamin-2 derivatives, hydrodamin-2-5, hydrodamin-2-6 and hydrodamin-2-10 showed the best activity (Supplementary Fig. 7o,p), although slightly less than the top performers from other groups. Hepserelin-3 derivatives, namely, hepserelin-3-4 and hepserelin-3-6, and arctoterin-1-5 were the strongest depolarizers in their respective families (Supplementary Fig. 7s,t). Equusin-4 derivatives, particularly equusin-4-2 and equusin-4-7, exhibited uniformly high depolarization activity (Supplementary Fig. 7a,b). Overall, the best depolarizers from each group displayed comparable efficacy, with hydrodamin-2 derivatives being slightly less potent than the rest. These results indicate that although many optimized peptides displayed measurable membrane permeabilization or depolarization, these effects did not consistently correlate with MIC values, suggesting that improvements in antimicrobial potency may arise from a combination of membrane and non-membrane-related mechanisms.

#### Cytotoxicity assays

All derivatives were tested for cytotoxic activity against human embryonic kidney (HEK293T) cells and compared with their templates (Fig. 3d). This assay is widely used to assess the toxicity of antimicrobials because the results are highly reproducible^31–33^. Of the peptides tested, 84 displayed no detectable cytotoxicity at the concentration range tested (4–64 μmol l^−1^). Among the equusin-4 derivatives, only 5, 6, 7 and 10 showed mild to moderate toxicity, whereas the most active derivatives were non-toxic. Arctoterin-1-1, the most active and broad-spectrum derivative, was the only cytotoxic peptide in its family. Interestingly, although the lophiosin-1 template was cytotoxic, all its derivatives were non-toxic. Most mylodonin-2 derivatives were non-toxic except for mylodonin-2-4 at high concentrations.

Mylodonin-3 derivatives optimized for broad-spectrum activity exhibited higher toxicity compared with those optimized for Gram negatives, with mylodonin-3-9 and mylodonin-3-10 being the most toxic, though still at concentrations 10 to 20 times higher than their MICs. Among the mammuthusin-2 derivatives, only mammuthusin-2-5, which had lower antimicrobial activity, was toxic. Mammuthusin-3 and hydrodamin-3 derivatives were non-toxic within the tested concentration ranges. Hydrodamin-2 derivatives with double or triple tryptophan substitutions, optimized for broad-spectrum activity, showed higher toxicity than those targeting Gram negatives. Hesperelin-3 derivatives were generally non-toxic, except for hesperelin-3-9, which had a MIC five to ten times lower than its cytotoxic concentration. Overall, the cytotoxicity varied across peptide families and was often related to the specific optimizations made for antimicrobial activity.

#### Resistance to proteolytic degradation assays

Proteolysis assays in human serum are resource-intensive and consume relatively large quantities of peptide. Before conducting in vivo mouse experiments, we assessed the stability of the parent peptides mammuthusin-3 and mylodonin-2, as well as their most active and non-toxic derivatives, mammuthusin-3-6 and mylodonin-2-3, in the presence of serum proteases. Interestingly, after 2 h of incubation, approximately 40% of mylodonin-2-3 remained intact. We attribute this enhanced stability to the replacement of the original arginine residues in mylodonin-2 with aliphatic isoleucine residues, which are less susceptible to proteolysis. By contrast, mammuthusin-3-6 was degraded within the first 30 min, most probably because an arginine residue was introduced relative to the parent peptide (Supplementary Fig. 8).

#### Anti-infective efficacy of optimized peptides in animal models

To evaluate the in vivo anti-infective efficacy of the most active optimized derivatives from two highly active template peptides, we used two preclinical mouse models: skin abscess^33–36^ and intramuscular thigh infection^36–38^. Two de-extinct compounds active against *A. baumannii* and their respective most active, non-toxic AI-optimized derivatives were tested with a single dose at their MIC concentration after the infection was established. The peptides had a wide range of MIC values (16–64 μmol l^−1^) when tested in vitro against *A. baumannii*: mammuthusin-3 and mammuthusin-3-6 (MIC values of 32 and 16 μmol l^−1^, respectively) from the woolly mammoth *Mammuthus primigenius* and hydrodamin-3 and hydrodamin-3-9 (MIC values of 16 and 8 μmol l^−1^, respectively) from the extinct manatee *Hydrodamalis gigas*; and mylodonin-2 and mylodonin-2-3 (MIC values of 64 and 16 μmol l^−1^, respectively) from the extinct giant sloth *Mylodon darwinii*.

**Fig. 4:**
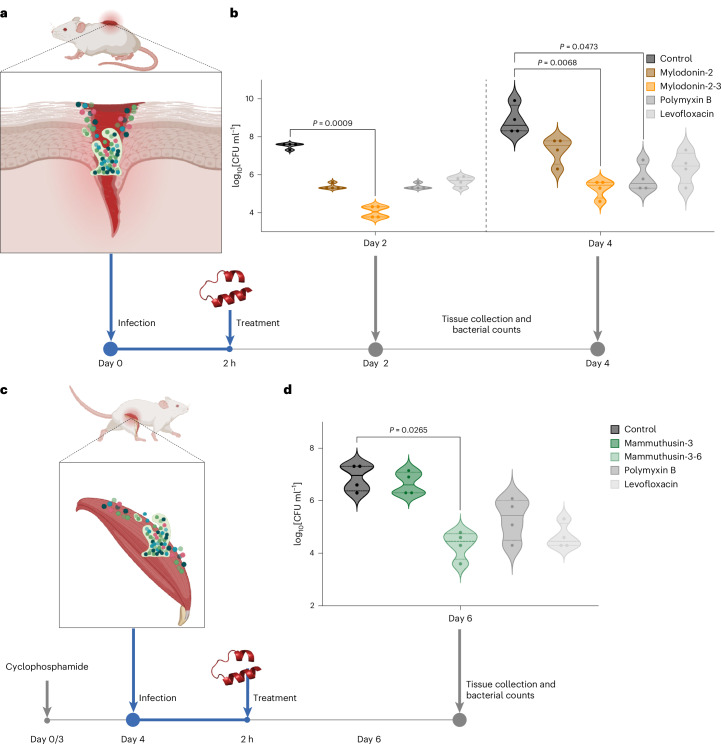
Anti-infective activity of templates and ApexGO-optimized peptides in animal models. **a**, Schematic of the skin abscess mouse model used to assess the anti-infective activity of the designed peptides against *A. baumannii* ATCC 19606. **b**, Comparison of the antibacterial efficacy between the parent de-extinct peptide mylodonin-2 and its most active, non-toxic ApexGO-optimized derivative, mylodonin-2-3. Peptides were administered as a single dose at their respective MICs post-infection (*n* = 4 per group). Two days after infection, the optimized peptide—mylodonin-2-3—significantly reduced bacterial loads by four orders of magnitude compared with the untreated control, outperforming the antibiotics by one order of magnitude. After 4 days, the optimized peptide exhibited higher anti-infective activity than its parent peptides, maintaining efficacy equivalent to the control antibiotics. **c**, Schematic of the deep thigh infection mouse model, where neutropenic mice were infected with *A. baumannii* ATCC 19606 and treated intraperitoneally with a single dose of mammuthusin-3 and its most active ApexGO-optimized derivative, mammuthusin-3-6 (*n* = 4 per group). **d**, Two days post-infection and treatment (day 6), mammuthusin-3-6 demonstrated significant antibacterial activity, reducing bacterial counts by three orders of magnitude compared with the untreated control group and matching the efficacy of the positive control antibiotics. Statistical significance in **b** and **d** was determined using one-way ANOVA followed by Dunnett’s test; *P* values are indicated in the graphs. In the violin plots, the centre line represents the mean, the box limits indicate the first and third quartiles, and the whiskers (minima and maxima) extend to 1.5× the interquartile range. Illustrations created in BioRender; **a**, de la Fuente-Nunez, C. http://biorender.com/n1k446g (2026); **c**, de la Fuente-Nunez, C. http://biorender.com/n1k446g (2026).

In the skin abscess infection model, mice were infected with a bacterial load of 5 × 10^5^ cells of *A. baumannii* (Fig. 4a). Each peptide or antibiotic (positive control) was administered as a single dose at their MIC over the infected area. After 2 days, the bacterial counts revealed that all the tested peptides significantly reduced the bacterial load by up to four orders of magnitude. In particular, mylodonin-2-3 demonstrated faster bacterial clearance in the skin abscess model compared with all controls. By day 2 post-treatment, colony-forming units (CFU) levels in the mylodonin-2-3 group were markedly reduced relative to polymyxin B, levofloxacin and the parent peptide, indicating a strong therapeutic response at an early time point. These findings underscore the potent anti-infective properties of these molecules against *A. baumannii* infections and the efficacy of our ApexGO optimization model in generating high-activity peptides. After 4 days, the optimized derivative mylodonin-2-3 exhibited slightly higher activity than their parent compound, showing antibacterial efficacy similar to that of widely used antibiotics polymyxin B and levofloxacin (Fig. 4b). No variations in weight, damage to the skin tissue, or other harmful consequences induced by the molecules or their derivatives were observed in the mice throughout our experiments (Supplementary Fig. 9a).

In the murine deep thigh infection model, the efficacy of mammuthusin-3-6 and its template was assessed following the establishment of an intramuscular infection (Fig. 4c). This well-established preclinical model is suited for evaluating the translatability of potential antibiotics. Mice were rendered neutropenic with cyclophosphamide before intramuscular injection of 9 × 10^5^ *A. baumannii* cells (Fig. 4d). A single dose of each compound at its MIC was administered intraperitoneally. Two days post-treatment, mammuthusin-3-6 demonstrated activity comparable with the positive control antibiotics, polymyxin B and levofloxacin, reducing the bacterial counts by three orders of magnitude. Four-days post-treatment, the peptides showed bacteriostatic activity reducing the bacterial load by two orders of magnitude compared with the untreated control group (Fig. 4d). No toxicity was observed in treated mice, as indicated by stable weight monitoring (Supplementary Fig. 9b).

These robust in vivo results demonstrate that the optimized derivatives possess potent anti-infective efficacy under physiologically relevant conditions. The optimized peptides not only improved on their template counterparts but also performed comparably with widely used antibiotics, highlighting their potential as effective antimicrobial agents.

## Conclusion

This study demonstrates the power of integrating generative AI approaches for antibiotic optimization. We present ApexGO, which successfully enhanced the activity of peptides mined from extinct organisms and identified derivatives with potent antimicrobial efficacy against high-priority bacterial pathogens, including multidrug-resistant strains. The optimized compounds exhibited favourable in vitro and in vivo profiles, with some outperforming conventional antibiotics in preclinical models. Among the peptides tested in vivo, mylodonin-2-3 showed the most striking improvement, achieving rapid bacterial clearance in the skin abscess model. The significant reduction in bacterial burden by day 2 relative to both standard-of-care antibiotics and the parent template highlights the potential of ApexGO to generate optimized leads with accelerated in vivo efficacy. This result underscores the value of applying our optimization approach to peptide templates.

Our findings underscore the potential of combining deep learning and BO to accelerate antibiotic discovery. ApexGO represents a substantial advancement in peptide design, streamlining the optimization process and opening promising avenues for developing antibiotics and other therapeutics. Translating antimicrobial agents to clinical use involves performing chemical modifications and optimizing properties beyond antimicrobial activity, such as stability, bioavailability and safety. In future work, we will focus on extending ApexGO to include multiproperty optimization, because linear AMPs typically exhibit greater proteolytic susceptibility and shorter half-life than cyclic lipopeptides, our comparisons with the positive-control antibiotic are intended solely as contextual benchmarks, not therapeutic parity claims. Subsequent cycles will pair ApexGO potency gains with stability/pharmacokinetics engineering (for example, end-capping, D-residues, lipidation, PEGylation or cyclization). Building on the APEX predictor, which performs strain-specific antimicrobial predictions against 11 pathogen strains, ApexGO enables objective adjustment to target specific subsets of these strains, allowing for the generation of AMPs with tailored strain selectivity. To further promote cross-strain gains and avoid unintended spectrum shifts, future iterations will use multiobjective constraints (regularizing physicochemical properties such as hydrophobic moment and net charge) and species-aware oracles. Nevertheless, due to the absence of explicit pathogen information in the model, the current APEX cannot be directly applied to pathogen strains beyond the original 11 used in training. A future enhancement will involve incorporating pathogen-specific features—such as genomic information—as additional inputs to enable APEX to perform transferable AMP prediction for other pathogens via zero-shot or few-shot learning. With this augmentation, ApexGO will be capable of designing effective AMPs against any bacterial species. Because ApexGO’s search is driven entirely by the APEX oracle, interpreting ApexGO designs reduces to understanding which sequence features APEX uses to assign a low predicted MIC. As an initial step, we extracted and analysed APEX attention weights for representative optimized peptides and across the top-performing design cohort (Supplementary Figs. 10 and 11). More comprehensive explainability, for example, integrating attribution methods beyond attention and connecting these patterns to experimentally validated biophysical mechanisms, remains an important direction for future work.

## Methods

### High-level optimization summary

We include here a high-level summary of our optimizer and architecture, the exact details of which we provide below. ApexGO consists of three components. The first component is a generative model that can produce peptides. We train our generative model broadly over large sets of protein and peptide space drawn from UniProt to be capable of producing arbitrary, even non-AMPs. The second component is an oracle—in our case, the APEX model described below—that predicts the MIC (in μmol l^−1^) of any input peptide against 11 different bacterial pathogens. Finally, we use an optimization algorithm—particularly, we develop a BO approach that extends recent work in this area—to induce the generative model to produce peptides that APEX will score highly. The resulting peptides we find that APEX predicts have low MIC are the ‘optimized’ peptides that we characterize experimentally.

### APEX 1.1

APEX^8^ is a deep learning-based model that takes peptide sequences as inputs and predicts MIC against *A. baumannii* ATCC 19606, *E. coli* ATCC 11775, *E. coli* AIC221, *E. coli* AIC222, *K. pneumoniae* ATCC 13883, *P. aeruginosa* PAO1, *P. aeruginosa* PA14, *S. aureus* ATCC 12600, methicillin-resistant *S. aureus* ATCC BAA-1556, vancomycin-resistant *E. faecalis* ATCC 700802 and vancomycin-resistant *E. faecium* ATCC 700221. APEX 1.1 (ref. ^39^) used a hybrid of recurrent and attention neural networks to extract peptide-level features that are useful for antimicrobial prediction from physicochemical and biomedical properties of amino acid sequences and was jointly trained by publicly available AMPs^10–12^ and our internal peptide antimicrobial activity data. Through wet-laboratory experimental validation, we demonstrated the reliability of APEX 1.1 on finding AMPs. In the ApexGO framework, APEX 1.1 serves as the oracle function and provides the MICs of peptides to guide the BO to propose peptide sequences with improved antimicrobial activities. For the rest of the Article, we refer to this oracle as APEX.

### Black-box BO

In black-box optimization, we aim to optimize an oracle objective function $f\left(x\right)$ over a space of candidates ${x}^{* }={\mathrm{argmax}}_{x\in {\mathcal{X}}}f\left(x\right)$. Examples of such problems include maximizing the binding affinity of small molecules^19,40^ or proteins^41,42^. Commonly, $f\left(x\right)$ is assumed to be expensive to evaluate and accessible only through evaluation, that is, the underlying behaviour of the objective is unknown.

BO is a sample-efficient model-based framework to solve these costly to evaluate optimization problems^43–45^. At iteration ${t}$ of BO, one has access to observations ${D}_{t}={\left\{\left[{x}_{i},{y}_{i}\,\right]\right\}}_{i=1}^{t}$, where ${y}_{i}$ denotes the (possibly noisy) objective value of the input ${x}_{i}$. Typically, a Gaussian process^46^ or approximate Gaussian process^23,47^ is used as the surrogate model to approximate the objective function using these inputs and values. This surrogate model aids the optimization by using an acquisition function, which utilizes the surrogate model’s understanding of the objective to strategically propose the next candidates for evaluation. After querying these candidates through the true oracle, the surrogate model is updated with the observations. This process gradually builds a more comprehensive dataset and refines the surrogate model, thereby improving the quality of the proposed samples in future iterations.

### BO over latent spaces

Due to the discrete and structural nature of peptide sequences, we utilize recent developments in latent space BO that adapt BO from continuous black-box optimization problems to the discrete domain^48,49^. Latent space BO leverages the capabilities of deep generative models, most commonly VAEs^21^ to aid optimization. Concretely, a VAE is composed of two networks: an encoder *E*(*z*|*x*):*X*→*P*(*Z*) mapping from amino acid sequences to a latent space $Z$, and a decoder *D*(*x*|*z*):*Z*→*P*(*X*) that probabilistically decodes latent space vectors back into amino acid sequences *X*. The VAE is trained in a self-supervised fashion on a large set of unlabelled amino acid sequences using the following standard VAE loss:

$${{\mathcal{L}}}_{\mathrm{VAE}}={{\mathbb{E}}}_{{\mathcal{E}}\left(Z,|,X\right)}\left[\log [D\left(X,|,Z\right)]\right]-\beta {KL}{\mathscr{(}}{\mathcal{E}}\left(Z,|,X\right)\mathrm{||}p(Z)).$$

The first term encourages reconstruction accuracy (the amino acid sequences we pass into the encoder being the same as the one we get out of the decoder), and the second Kullback–Leibler divergence term encourages smoothness of the latent space by regularizing the encoder towards the prior distribution $p(Z)\triangleq {\mathscr{N}}(0,{\bf{I}})$. In this work, we train a six-layer transformer encoder and decoder VAE that bottlenecks down to two tokens with 128 latent dimensions each, for a total of 256 latent dimensions. We train with a Kullback–Leibler regularization factor of $\beta ={10}^{-4}$.

Sequences are tokenized as 1-mers (*k* = 1) using a vocabulary of the 20 standard amino acids plus special tokens <start>, <stop> and the pad –. We append <stop> during encoding and pad to batch length with the <stop> index. Decoding prepends the <start> token; the sampler treats index 0 as <start> and index 1 as <stop> and generates until the first <stop> or a length cap. For generation, we set max_string_length = 50. We trained with Adam (PyTorch defaults) at lr = 2 × 10^−4^, batch size = 512, mixed precision (bfloat16 autocast) with GradScaler, and no weight decay or gradient clipping. We validated every three epochs and saved the best-loss checkpoint. Data splits were 90/5/5 for the train/validation/test sets. Unless noted, all other hyperparameters follow the defaults in the training script (encoder/decoder feed-forward size = 256; dropout = 0.05).

After the VAE is pretrained on a large number of peptide sequences, for example, all sequences in UniRef^50^ within a certain size range, we can define our search over the continuous latent space *Z* of the VAE instead of the discrete space of amino acid sequences *X*, we can now formulate our optimization problem as

$${x}^{\star }{\mathscr{\approx }}{\mathcal{D}}\left({z}^{\star }\right),\,\mathrm{where}\,{x}^{\star }={\mathrm{argmax}}_{z{\mathscr{\in }}{\mathcal{Z}}}f\,{\prime} (D(z)).$$

The objective function we are optimizing now takes in a latent vector $z$ and decodes it into an amino acid sequence, which is then evaluated on our black-box objective function *f*. Because the true objective function (that is, MIC) requires expensive laboratory synthesis to evaluate, we run optimization using our APEX model *f*′, which estimates the true MIC of the amino acid sequence. After running optimization to obtain sequences that achieve low MICs according to APEX, we then select a batch of the best-performing sequences that are then sent to the laboratory for synthesis and experimental validation. Our optimization algorithm is based on our prior work, the LOL-BO algorithm^19^, which we adapt here for the constrained template derivative optimization setting.

Before beginning optimization, we pretrained a VAE model on unlabelled amino acid sequences. We train a VAE on 4.5 million amino acid sequences of less than 50 amino acids in length, randomly cropped from the UniRef database^50^ for 118 epochs on a single NVIDIA RTX A6000 GPU to obtain a final test-set reconstruction accuracy of 99.94%. We reused this same initial pretrained VAE for all optimization returns, with some additional unlabelled fine tuning, as described below.

We then ran BO in the latent space of the VAE model to find amino acid sequences that minimize $f\,{\prime} \,(D(z))$, where $f\,{\prime}$ was the estimated MIC of the amino acid sequence according to our APEX model. We use a parametric Gaussian process regressor surrogate with 1,024 inducing points to model the data collected during optimization^23^, and select the best candidate latent space point $z$ to evaluate next on each iteration of optimization using Thompson sampling to perform acquisition.

Following the LOL-BO algorithm^19^, we also periodically update our VAE model jointly and in an end-to-end fashion with the GP surrogate model to encourage the VAE latent space to reorganize such that peptides with similar scores (MICs) are moved closer together and the space becomes more ideal for modelling and optimization. The models are updated jointly using the following loss:

$${{\mathcal{L}}}_{\mathrm{joint}}={{\mathbb{E}}}_{{\mathcal{E}}{\mathscr{(}}{Z|X})}\left[{{\mathcal{L}}}_{\mathrm{PPGPR}}\left(y,\,Z\right)\right]+{{\mathcal{L}}}_{\mathrm{VAE}}(X).$$

Here the first term is an expectation over the encoder of the typical^36^ parametric Gaussian process regressor loss ${{\mathcal{L}}}_{\mathrm{PPGPR}}(y,Z)$, and ${{\mathcal{L}}}_{\mathrm{VAE}}$ is the VAE loss described above.

### Using trust regions

In addition to the joint model updates^19^, it was shown that latent space optimization can be improved by using trust region optimization to limit over-exploration in the very high-dimensional latent search space. We, therefore, use trust region BO^24^. This method works by defining a hyper-rectangular trust region within the search space, and limits the search for candidates to this local region. The trust region is always defined to be centred on the best point observed so far (the latent space point that achieved the best score $y=f\,{\prime} (D(z))$ we have seen so far), and the size of the trust region is adapted on each iteration depending on the recent success of the optimizer, with the initial trust region width set to 0.8. If we fail to make progress (to find a latent point *z* that achieves a better score than the best we have seen so far) for 32 consecutive optimization iterations, the length of the trust region is reduced by a factor of two to further limit overexploitation. Conversely, when we do make progress for ten consecutive iterations, the length of the trust region doubles to allow more exploration.

### Adding constraints

A strong desideratum when optimizing each template peptide was to maintain high similarity to the initial template, we choose this similarity constraint because we want to obtain the derivatives of peptides that were previously validated, that is, similarity of >75%. We, therefore, optimize under an added constraint that all amino acid sequences proposed by the optimization algorithm must be at least 75% similar to the template sequence. Similarity here is measured as $(l-d)/l$, where $l$ is the length of the seed peptide and $d$ is the Levenshtein distance between the proposed peptide and the seed.

To add this constraint, we adapted the scalable constrained BO algorithm^51^ to the latent space/generative BO setting. This algorithm works by training a second GP surrogate to model the constraint function $C(D(z))$. In our case, *C*(*D*(*z*)) is the similarity of the proposed peptide *D*(*z*) to the template sequence, as measured by the fraction of sequence overlap. During optimization, we require that *C*(*D*(*z*)) ≥ 75% for all generated candidates *z* proposed by the optimizer. On each iteration of optimization, when we use Thompson sampling to select a candidate point *z* to evaluate next, we exclude all sampled *zs* for which the constraint GP model predicts that the sample will be infeasible (that is, *C*(*D*(*zs*)) < 75%). Then, for each remaining candidate latent vector *z*, we evaluate the true constraint function *C*(*D*(*z*)) and update the constraint GP.

### Fine-tuned VAEs for template optimization

Different from the setting considered in the original LOL-BO, we seek to specifically produce optimized derivatives of pre-existing templates. However, VAEs trained on global sets of proteins will devote only small regions of the latent space to producing sequences with high similarity to any particular template: in other words, most sequences produced by the generative model a priori would look nothing like the template. To bias decoding towards a given template and maintain sequence closeness, we generate 20,000 synthetic derivatives by random mutagenesis within the similarity constraint used downstream (≥75% identity by edit distance to the template). We then continue training the pretrained VAE on this per-template derivative set using the same optimizer, learning rate (2 × 10^−4^), batch size (512), dropout (0.05), *β* (1 × 10^−4^) and model dimensions as in pretraining. This increases the rate at which the VAE produces candidates that satisfy the hard similarity constraint imposed during BO, without changing the APEX-guided objective. The mutation generator and fine-tuning scripts are available in our GitHub repository (see the code availability statement) to support full reproducibility. This has the effect of notably increasing the rate at which the VAE produces sequences similar to the template. Although the generated sequences will not initially improve antimicrobial activity, this improves gradually over the course of optimization because we update the VAE jointly with the surrogate model.

### Using multiple trust regions to optimize a set of multiple sequences

Rather than finding a single amino acid sequence that achieves low MIC according to the APEX model, what we want to do is find a set of unique amino acid sequences that all achieve low MIC according to APEX. Having a set of different sequences is important because it increases the chance that some of them will achieve success when they are sent to the wet laboratory for validation. Thus, rather than searching for a single optimal amino acid sequence, we simultaneously optimize a set of *M* = 20 unique amino acid sequences. To accomplish this, we follow ROBOT^20^ in using 20 different trust regions centred on the 20 unique best-scoring points found so far. Each trust region is responsible for finding one of the 20 final unique sequences in our final set of optimal sequences. On each step of optimization, we select candidate points from within each of the 20 trust regions. In the *i*th trust region, we discard and resample candidates that are insufficiently diverse from the candidates chosen by trust regions *j* < *i* according to our chosen diversity metric. Specifically, in ApexGO, we discard candidates from the *i*th trust region if any early trust region has already proposed that candidate exactly. We update the size of each trust region according to that individual trust region’s success or failure to propose a sequence that improves on the best-scoring sequence in that trust region. The 20 trust regions use a shared data history and are recentred after each iteration so that they are always centred on the best 20 unique sequences observed so far. At the end of the optimization, our final set of optimized sequences are the final centres of the 20 trust regions.

### Optimization goal and setup

During optimization, we aim to produce peptides that achieve low average predicted MIC according to the APEX 1.1 model. We perform separate optimization runs against two objectives: (1) the average predicted MIC against Gram-negative bacteria only (that is, *A. baumannii* ATCC 19606, *E. coli* ATCC 11775, *E. coli* AIC221, *E. coli* AIC222, *K. pneumoniae* ATCC 13883, *P. aeruginosa* PAO1 and *P. aeruginosa* PA14), and (2) against both Gram-negative and Gram-positive bacteria (that is, *A. baumannii* ATCC 19606, *E. coli* ATCC 11775, *E. coli* AIC221, *E. coli* AIC222, *K. pneumoniae* ATCC 13883, *P. aeruginosa* PAO1, *P. aeruginosa* PA14, *S. aureus* ATCC 12600, methicillin-resistant *S. aureus* ATCC BAA-1556, vancomycin-resistant *E. faecalis* ATCC 700802 and vancomycin-resistant *E. faecium* ATCC 700221). In both cases, the BO minimized the objectives (that is, lower MICs corresponds to higher antimicrobial activities). In addition to the minimization objective, we used a similarity constraint in which each generated peptide should be at most $25 \%$ different to given template, where difference is measured as $\frac{{\rm{distance}}}{{l}_{{\rm{template}}}}$. We used a batch size of 50 for optimization, with 20 trust regions and *τ* = 1, meaning that we aim to produce 20 different peptides for the same template in a single optimization run. We update our surrogate model and VAE jointly every ten steps of optimization and initialize the optimization with 10,000 peptides randomly mutated from the template peptide.

### Enumeration of mammuthusin-3 and arctoterin-1

Two of our parent template peptides, mammuthsin-3 (10 residues long) and arctoterin-1 (15 residues long), are sufficiently short such that under our similarity constraint of 75% by edit distance, it is feasible to enumerate and score all the possible derivative peptides using the APEX oracle. This comprehensive approach allowed us to directly compare ApexGO with the highest-scoring derivative peptides. In particular, for both templates, ApexGO recovered all 20 of the top-20 enumerated peptides, indicating that the derivatives validated in this study were globally optimal according to the APEX oracle. This result highlights the strength of our optimizer, as ApexGO successfully identified the best possible peptides (according to APEX) in both settings in which it was computationally feasible to verify this. This result highlights the strength of our optimizer: whenever the search space can be fully enumerated, ApexGO still lands on every global optimum. By contrast, a 20-residue template that permits up to five single-site edits (substitution, insertion or deletion) already spans ~3 × 10^12^ unique sequences; even screening a million designs per minute would take more than 1,000 years to exhaust that space. Because exhaustive search is infeasible for many realistic peptide design tasks, the utility of ApexGO lies in its ability to rapidly discover multilog improvements within a realistic experimental budget—demonstrated here by its perfect recovery of all top-ranked variants for short templates and, for longer peptides, by routinely achieving 8–16-fold gains in MICs and avoiding impractical brute-force evaluation.

### Benchmark against existing BO frameworks

To further highlight our approach, we benchmarked ApexGO against state-of-the-art generative BO approaches from the literature. In Supplementary Fig. 11, we compare ApexGO directly with (1) the LOL-BO implementation^19^ and (2) a standard latent space BO pipeline, adapted with the high-dimensional trust region heuristics^51^. To ensure fair comparison, all methods were evaluated using the same peptide VAE with fixed weights. For each method, we optimized all ten template peptides and plotted the APEX-predicted MIC of the best peptide returned by each run (ten data points per method; Supplementary Fig. 12). ApexGO outperforms both baselines across all ten templates: the worst-performing ApexGO run matches the best run of standard BO, and LOL-BO is consistently outperformed by ApexGO despite using a comparable closed-loop framework. These results confirm that ApexGO’s joint VAE-retraining, multiple-trust-region strategy delivers state-of-the-art performance in peptide design.

### Benchmark against HydrAMP and PepDiffusion

To compare ApexGO against contemporary deep generative AMP models, we evaluated HydrAMP^16^ and the PepDiffusion^17^ latent diffusion model for derivative generation using the same ten extinct template AMPs that serve as seeds for ApexGO in the main experiments.

We used the official HydrAMP implementation and the publicly released generator and decomposer weights. For each seed sequence, we called the analogue_generation routine under the discovery filtering criterion with decoder temperature $T\in \{\mathrm{3,5,10}\}$, using batches of 256 attempts and a fixed proposal budget of at most 1,000,000 total candidates and 200,000 unique sequences per seed. HydrAMP proposes derivatives without the explicit knowledge of our similarity constraint; therefore, after generation, we computed the Levenshtein distance between each candidate and the corresponding seed and retained only those with similarity of ≥75%, defined as $1-\,\frac{{\rm{distance}}}{{l}_{{\rm{template}}}}$. The surviving derivatives were scored with the same APEX Gram-negative objective used by ApexGO (lower values indicate better predicted activity). For each seed and temperature, we then selected the top-*K* feasible sequences, where *K* equals the number of ApexGO designs produced for that seed, and compared their objective values with ApexGO’s top-*K* set (Supplementary Fig. 1).

To evaluate panel-level robustness, we calculated per-organism predicted MIC distributions across all seven Gram-negative targets. Here 84.9% of ApexGO’s designs improved predicted MIC relative to the seed across all seven Gram-negative organisms, compared with 50.3%, 55.7% and 32.9% for HydrAMP at decoder temperatures of *T* = 3, 5 and 10, respectively. The median worst-case predicted Gram-negative MIC across seeds is 108.8 μmol l^−1^ for ApexGO, versus 210.6 μmol l^−1^ (*T* = 3), 192.1 μmol l^−1^ (*T* = 5) and 302.3 μmol l^−1^ (*T* = 10) for HydrAMP (Supplementary Figs. 13 and 14). To further assess HydrAMP in a ground-truth experimental setting, we synthesized the top-two HydrAMP-generated analogues for each of the ten seed templates (20 peptides in total; we were not able to synthesize one of the selected peptides using solid-phase peptide synthesis) and measured their MIC values against the same panel of 11 clinically relevant pathogens used to evaluate ApexGO. HydrAMP’s performance was highly seed dependent. For templates whose sequences resemble canonical AMPs, well represented in HydrAMP’s training set, which were short, cationic, leucine/lysine-rich scaffolds such as mammuthusin-3 (KTLKIIRLLF, ten amino acid residues), mylodonin-2 (KRKRGLKLATALSLNNKF, 18 residues long) and mammuthusin-2 (26 amino acid residues, arginine rich), HydrAMP produced potent derivatives, with HydrAMP-12 and HydrAMP-14 achieving MICs as low as 1 μmol l^−1^ against *A. baumannii* and *E. coli* strains (Supplementary Fig. 15). However, for templates with sequence motifs atypical of known AMPs, particularly the de-extinct peptides hesperelin-3 (RQKNHGIHFRVLAKALR, containing the unusual HGIH motif) and lophiosin-1 (HWITINTIKLSISLKI, with an N-terminal HW-TINT motif), both HydrAMP derivatives were completely inactive (MIC > 64 μmol l^−1^ against all strains tested). A similar pattern was observed for hydrodamin-2 (RMARNLVRYVQGLKKKKVI), where HydrAMP derivatives showed only marginal activity (MIC, 32–64 μmol l^−1^, against three out of seven Gram-negative strains). This seed-dependent performance is consistent with a distribution mismatch: HydrAMP’s conditional VAE, trained on curated AMP databases, generates derivatives that conform to the sequence patterns of known AMPs and struggles to optimize scaffolds that fall outside this learned distribution. By contrast, ApexGO produced experimentally active derivatives (MIC ≤ 64 μmol l^−1^ against ≥3 Gram-negative strains) for all ten templates, including hesperelin-3 and lophiosin-1, because its BO loop guided by the APEX oracle does not rely on the generative model having previously encountered similar sequences during training, only on the oracle’s ability to score proposed edits. These results illustrate a fundamental distinction between one-shot generative approaches, which are effective within their training distribution, and optimization-based frameworks like ApexGO that can navigate unfamiliar sequence neighbourhoods through iterative, feedback-driven search (Supplementary Fig. 15).

PepDiffusion combines a transformer-based VAE with a conditional latent diffusion model trained on AMP and non-AMP sequences, followed by classifier- and MD-based filtering. As a baseline for our derivative design task, we used the best-performing VAE and conditional diffusion checkpoints and their default sampling hyperparameters (500 diffusion steps with a maximum length of 50), conditioning on the AMP class label (cond_label = 0). We generated 1,000,000 unique candidate sequences in this manner. Similar to HydrAMP, we then computed the Levenshtein-distance-based similarity between each candidate and each of our ten seed templates and filtered to those with similarity of $\ge 75 \%$ to at least one seed. In this constrained task, PepDiffusion did not produce any sequences above the similarity threshold for any seed, so no top-*K* derivative sets could be formed, and no further objective comparison was possible. This outcome is consistent with the model’s intended use for template-free, diversity-oriented AMP discovery rather than per-template-constrained optimization.

### Sequence space exploration

Our primary objective was to identify peptides with enhanced antimicrobial activity. Accordingly, our experimental approach in this study focuses chiefly on modifying template peptides from extinct animals. To evaluate the capacity of ApexGO to navigate the full sequence search space, we performed optimization and completely relaxed the similarity constraint, optimizing peptides for four different objectives: peptides generated with 0% similarity constraint (Free), peptides with predicted broad-spectrum activity and 0% similarity constraint (Broad-free), peptides with at least 75% dissimilarity to known AMPs (Free-75%diff) and peptides with at least 75% dissimilarity to known AMPs and predicted broad-spectrum activity (Broad-free-75%diff). This approach allowed the optimizer to generate peptides without restriction to template sequences (Supplementary Table 2). These similarity levels (typically 40%–60%) fall below the ≥70% identity threshold commonly used to define analogues, reinforcing that ApexGO-generated peptides are distinct. The resulting peptides exhibited antimicrobial activity, with some displaying potency at very low concentrations (1–2 μmol l^−^^1^) (Supplementary Fig. 16). In particular, the optimizer generated peptides that are highly dissimilar to the original templates, as quantified by the edit distance (Supplementary Fig. 17).

### Striped Smith–Waterman alignment-based similarity score

We use ${\rm{SSW}}(a,b)$ to denote the optimal alignment score of protein sequences $a$ and $b$ using the Striped Smith–Waterman algorithm (SSW)^52^. The SSW similarity score can then be defined as $\frac{{\rm{SSW}}(a,b)\,}{\sqrt{{\rm{SSW}}\left(a,a\right)\times {\rm{SSW}}(b,b)\,}}$.

### Peptide synthesis

All peptides for the experiments were obtained from AAPPTec and synthesized using solid-phase peptide synthesis with the Fmoc strategy. All peptides were de-salted and determined to be >90% pure by high-performance liquid chromatography coupled to mass spectrometry analysis.

### Culturing conditions and bacterial strains

In this study, we used the following pathogenic bacterial strains: *A. baumannii* ATCC 19606, *E. coli* ATCC 11775, *E. coli* AIC221 [*E. coli* MG1655 phnE_2::FRT (control strain for AIC 222)], *E. coli* AIC222 [*E. coli* MG1655 pmrA53 phnE_2::FRT (polymyxin resistant; colistin-resistant strain)], *K. pneumoniae* ATCC 13883, *P. aeruginosa* PAO1, *P. aeruginosa* PA14, *Staphylococcus aureus* ATCC 12600, *S. aureus* ATCC BAA-1556 (methicillin-resistant strain), *Enterococcus faecalis* ATCC 700802 (vancomycin-resistant strain), and *Enterococcus faecium* ATCC 700221 (vancomycin-resistant strain). Pseudomonas Isolation (*P. aeruginosa* strains) agar plates were exclusively used in the case of *Pseudomonas* species. All the other pathogens were grown in Luria–Bertani (LB) broth and on LB agar. In all the experiments, bacteria were inoculated from one-isolated colony and grown overnight (16 h) in a liquid medium at 37 °C. On the following day, inoculums were diluted 1:100 in fresh media and incubated at 37 °C to mid-logarithmic phase.

### MIC assays

Broth microdilution assays were conducted to establish the MIC for each peptide^53–55^. Peptides were added to untreated polystyrene 96-well microtitre plates and serially diluted twofold in sterile water, ranging from 0 to 64 μmol l^−1^. A bacterial inoculum at a concentration of 10^6^ CFU ml^−1^ in LB medium was then mixed in a 1:1 ratio with the peptide solution. The MIC was determined as the lowest peptide concentration that completely inhibited bacterial growth after 24 h of incubation at 37 °C. Each assay was performed in three independent replicates.

### Circular dichroism experiments

The circular dichroism experiments were conducted using a J1500 circular dichroism spectropolarimeter (Jasco) in the Biological Chemistry Resource Center at the University of Pennsylvania. Experiments were performed at 25 °C, the spectra graphed are an average of three accumulations obtained with a quartz cuvette with an optical path length of 1.0 mm, ranging from 260 to 190 nm at a rate of 50 nm min^−1^ and a bandwidth of 0.5 nm. The concentration of all peptides tested was 50 μmol l^−1^, and the measurements were performed in water, a mixture of TFE and water in a 3:2 ratio, and SDS in water at 10 mmol l^−1^, with respective baselines recorded before measurement. A Fourier transform filter was applied to minimize the background effects. Secondary structure fraction values were calculated using the single spectra analysis tool on the server BeStSel^56^. Ternary plots were created in TernaryPlot.com (https://www.ternaryplot.com/) and subsequently edited.

### Outer membrane permeabilization assays

The NPN uptake assay was used to evaluate the ability of the peptides to permeabilize the bacterial outer membrane. Inocula of *A. baumannii* ATCC 19606 were grown to an optical density (OD) at 600 nm of 0.4 ml^−1^, centrifuged (9,391*g* at 4 °C for 10 min), washed and resuspended in 5 mmol l^−1^ of HEPES buffer (pH 7.4) containing 5 mmol l^−1^ of glucose. The bacterial solution was added to a white 96-well plate (100 μl per well) together with 4 μl of NPN at 0.5 mmol l^−1^. Consequently, peptides diluted in water were added to each well, and the fluorescence was measured at *λ*_ex_ = 350 nm and *λ*_em_ = 420 nm over time for 45 min. The relative fluorescence was calculated using the untreated control (buffer + bacteria + fluorescent dye) and polymyxin B (positive control) as baselines and the following equation was applied to reflect the percentage of difference between the baselines and the sample:

$$\begin{array}{l}{\rm{Percentage}}\,{\rm{difference}}\\ =\displaystyle \frac{100\times ({{\rm{fluorescence}}}_{{\rm{sample}}}-{{\rm{fluorescence}}}_{{\rm{untreated}}{\rm{control}}})}{{{\rm{fluorescence}}}_{{\rm{untreated}}{\rm{control}}}}\end{array}.$$

### Cytoplasmic membrane depolarization assays

The cytoplasmic membrane depolarization assay was performed using the membrane-potential-sensitive dye DiSC_3_-5. *A. baumannii* ATCC 19606 in the mid-logarithmic phase were washed and resuspended at 0.05 OD ml^−1^ (optical value at 600 nm) in HEPES buffer (pH 7.2) containing 20 mmol l^−1^ of glucose and 0.1 mol l^−1^ of KCl. DiSC_3_-5 (20 μmol l^−1^) was added to the bacterial suspension (100 μl per well) for 15 min to stabilize the fluorescence, which indicates the incorporation of the dye into the bacterial membrane, and then the peptides were mixed 1:1 with the bacteria to a final concentration corresponding to their MIC values. Membrane depolarization was then followed by reading changes in the fluorescence (*λ*_ex_ = 622 nm, *λ*_em_ = 670 nm) over time for 60 min. The relative fluorescence was calculated using the untreated control (buffer + bacteria + fluorescent dye) and polymyxin B (positive control) as baselines and the following equation was applied to reflect the percentage of difference between the baselines and the sample:

$$\begin{array}{ll}{\rm{Percentage}}\,{\rm{difference}}\\ =\displaystyle \frac{100\times ({\mathrm{fluorescence}}_{{\rm{sample}}}-{{\rm{fluorescence}}}_{{\rm{untreated}}{\rm{control}}})}{{{\rm{fluorescence}}}_{{\rm{untreated}}{\rm{control}}}}\end{array}.$$

### Cytotoxicity assays

One day before the experiment, an aliquot of 100 μl of the cells at 50,000 cells ml^−1^ was seeded into each well of the cell-treated 96-well plates used in the experiment. The attached HEK293T cells were then exposed to increasing concentrations of the peptides (8–128 μmol l^−1^) for 24 h. After the incubation period, we performed the 3-(4,5-dimethylthiazol-2-yl)-2,5-diphenyltetrazolium bromide tetrazolium reduction assay (MTT assay)^55^. The MTT reagent was dissolved at 0.5 mg ml^−1^ in the medium without phenol red and was used to replace cell culture supernatants containing the peptides (100 μl per well). The samples were incubated for 4 h at 37 °C in a humidified atmosphere containing 5% CO_2_, yielding the insoluble formazan salt. The resulting salts were then resuspended in hydrochloric acid (0.04 mol l^−1^) in anhydrous isopropanol and quantified by spectrophotometric measurements of absorbance at 570 nm. All assays were done as three biological replicates.

### Resistance to proteolytic degradation assay

The stability of the peptides against enzymatic degradation was assessed by incubation in a solution containing 25% human serum in water. Specifically, the peptides, at a concentration of 10 mg ml^−1^, were incubated with an aqueous solution of 25% human serum (Zen-Bio; healthy donor, blood type A–) for 6 h. Samples were collected at 0, 0.5, 1, 2, 4 and 6 h, and each aliquot was immediately treated with 10 μl of trifluoroacetic acid for 10 min to halt the enzymatic activity. The analyses were performed using a Waters Acquity ultrahigh-performance liquid chromatography–mass spectrometry system featuring a photodiode array detector (190–400-nm data collection) and a Waters single quadrupole detector 2. The setup included a Waters XBridge C_18_ column (3.5 µm, 4.6 mm × 50 mm), with a mobile phase consisting of 100% water containing 0.1% (v/v) formic acid (solvent A) and 100% acetonitrile (solvent B). Both solvents were of the Fisher Optima grade. A 50 μl of injection volume was used, and ionization was carried out in both positive and negative electrospray ionization modes, scanning a mass range of *m*/*z* 100–3,000. The gradient used consisted of 5%–95% solvent B for 5 min. The proportion of intact peptide remaining at each time point was calculated by integrating the area under the curve of the peptide peak at the initial time (*t* = 0) as a reference. The experiments were done in three replicates.

### Skin abscess infection mouse model

The back of six-week-old female CD-1 mice under anaesthesia were shaved and injured with a superficial linear skin abrasion made with a needle. An aliquot of *A. baumannii* ATCC 19606 (5 × 10^5^ CFU ml^−1^; 20 μl) previously grown in LB medium until 0.5 OD ml^−1^ (optical value at 600 nm) and then washed twice with sterile PBS (pH 7.4, 9,391*g* for 3 min) was added to the scratched area. A single dose of peptides or antibiotics (positive control groups) diluted in sterile water at their MIC value were administered to the wounded area 1 h post-infection. Two- and four-days post-infection, animals were euthanized, and the scarified skin was excised, homogenized using a bead beater (25 Hz for 20 min), tenfold serially diluted and plated on McConkey agar plates for CFU quantification. The experiments were performed using four mice per group. The skin abscess infection mouse model was revised and approved by the University Laboratory Animal Resources from the University of Pennsylvania (protocol number 806763).

### Deep thigh infection mouse model

Experiments were performed using six-week-old female CD-1 mice, which were rendered neutropenic by the intraperitoneal application of two doses of cyclophosphamide (150 mg kg^−1^ and 100 mg kg^−1^) 3 days and 1 day before the infection. On day 4 of the experiment, the mice were infected in their right thigh through a 100 μl of intramuscular injection of *A. baumannii* ATCC19606 (in PBS at a concentration of 9 × 10^5^ CFU ml^−1^). The bacterial cells were grown in LB broth, washed twice with PBS solution and diluted at the desired concentration before infecting the mice. Peptides or antibiotics (positive control groups) were administered as a single dose intraperitoneally 2 h after the infection. Four days post-infection, the mice were euthanized, and the tissue from the right thigh was excised, homogenized using a bead beater (25 Hz for 20 min), tenfold serially diluted and plated on McConkey agar plates for counting the bacterial colonies. The experiments were performed using four mice per group. The deep thigh infection mouse model was revised and approved by the University Laboratory Animal Resources from the University of Pennsylvania (protocol number 807055).

### Reporting summary

Further information on research design is available in the Nature Portfolio Reporting Summary linked to this article.

## Supplementary information

Supplementary Information Supplementary Figs. 1–17 and Tables 1 and 2.

Reporting Summary

Supplementary Data 1 Antimicrobial activity of templates and ApexGO-generated peptides against 11 relevant pathogenic strains.

## Data Availability

All data pertaining to the experimental validation of the generated peptides are available in Supplementary Data 1 and Mendeley Ddata (https://data.mendeley.com/datasets/kk53h8pt5v/1). Supplementary Data 1 is also available via Mendeley Data (https://data.mendeley.com/datasets/84hxrm2zsm/1).

## Code availability

The ApexGO model is available on GitHub (https://github.com/Yimeng-Zeng/ApexGO) and Zenodo (10.5281/zenodo.19241740)^57^.
