# Supplementary figures and images for: A generative artificial intelligence approach for peptide antibiotic optimization
Source: Nat Mach Intell. 2026 May 13;8(5):841–56. doi: 10.1038/s42256-026-01237-5 (PMC13201158; doi:10.1038/s42256-026-01237-5)

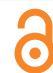

|   |                                |
|---|--------------------------------|
| 1 | <b>Supplementary Materials</b> |
| 2 | Supplementary Figs. 1 to 17    |
| 3 | Supplementary Tables 1 to 2    |

Supplement: Supplementary file 1 — Supplementary Figs. 1–17 and Tables 1 and 2. [file 42256_2026_1237_MOESM1_ESM.pdf]
